# Supplementary material for: Evidence supported by Mendelian randomization: impact on inflammatory factors in knee osteoarthritis
Source: Front Med (Lausanne). 2024 May 28;11:1382836. doi: 10.3389/fmed.2024.1382836 (PMC11165061; doi:10.3389/fmed.2024.1382836)
Supplement: Supplementary file 1 [file Data_Sheet_1.ZIP › data/GCST90274777_5e6/777.forest.pdf]

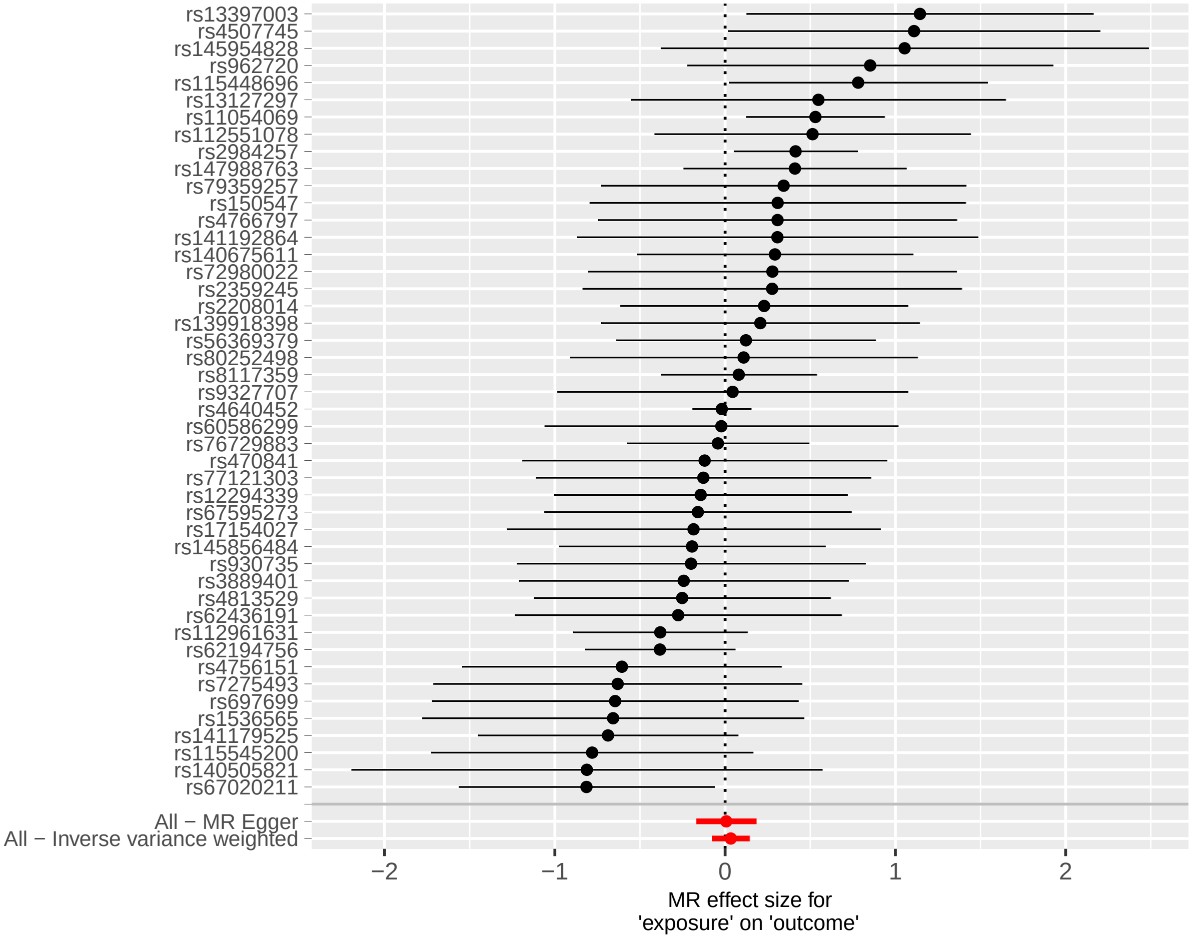
rs13397003
rs4507745
rs145954828
rs962720
rs115448696
rs13127297
rs11054069
rs112551078
rs2984257
rs147988763
rs79359257
rs150547
rs4766797
rs141192864
rs140675611
rs72980022
rs2359245
rs2208014
rs139918398
rs56369379
rs80252498
rs8117359
rs9327707
rs4640452
rs60586299
rs76729883
rs470841
rs77121303
rs12294339
rs67595273
rs17154027
rs145856484
rs930735
rs3889401
rs4813529
rs62436191
rs112961631
rs62194756
rs4756151
rs7275493
rs697699
rs1536565
rs141179525
rs115545200
rs140505821
rs67020211
All − MR Egger
All − Inverse variance weighted
−2
−1
0
1
2
MR effect size for
'exposure' on 'outcome'
